# A Multi-sectoral Approach to Capture Information on Road Traffic Injuries

**DOI:** 10.4103/0970-0218.66876

**Published:** 2010-04

**Authors:** Geetha R Menon, G Gururaj, MP Tambe, B Shah

**Affiliations:** Division of Non-Communicable Diseases, Indian Council of Medical Research, Ansari Nagar, New Delhi, India; 1 Department of Epidemiology, National Institute of Mental Health and Neurosciences (NIMHANS), Bangalore, India; 2 Department of Preventive and Social Medicine, Byramjee Jeejeebhoy Medical College (BJMC), Pune, Maharashtra, India

**Keywords:** Multi-sectoral, stakeholders, surveillance

## Abstract

**Background:**

Regularly available data is shown to be inadequate for developing, implementing, and evaluating injury prevention and control programs in India. The present study was undertaken in the hospitals of Bangalore and Pune, to examine the feasibility of gathering information on injuries using multiple sources.

**Materials and Methods:**

Stakeholders meeting and training programs were held for the hospital staff, police personnel, and traffic and transport staff, to identify their roles and responsibilities. Prospective data on morbidity and mortality due to injuries were collected by trained staff from Emergency Departments on a pre-tested questionnaire. The information gathered was cross-checked with the hospital and police records.

**Results:**

The stakeholders meeting and training programs were able to motivate the departments to provide the correct data. Data on 32188 patients could be extracted from hospital and police records during the study period. Injuries accounted for 16% of the emergency cases. Unintentional injuries were 64%, and 32% were intentional. Road traffic injuries accounted for 44% of all the injuries. One-third of the injured were children and young adults below 25 years. Among the injured, two wheeler riders were 29% and pedestrians were 23%.

**Conclusion:**

It was possible to improve the data on injuries by adequate training and a data linking mechanism between the Police, Hospital, and Transport Departments. The problem of road traffic injuries could be highlighted and addressed by a good data capture mechanism.

## Introduction

Injuries account for 12% of the global mortality, with more than five million deaths every year.(1) Among people, aged 15 to 29 years, eight out of fifteen deaths are injury-related. Deaths due to Road Traffic Injury (RTI) are the commonest cause among all injury deaths, killing 1.4 million people worldwide.(2) Also 20-50 million persons are left seriously injured due to RTIs annually. In the developing countries 85% of the total global mortality and 90% of the Disability Adjusted Life Years lost are due to RTIs. RTI accounts for 30 to 86% of the trauma admissions to hospitals in low income and middle income countries.(3) In addition, traffic injuries in these countries incur an annual loss of $65 billion to $100 billion annually. These costs include both loss of income and the burden placed on families to care for their injured relatives. If the current trend continues, RTIs will be the third leading contributor to the global burden of disease by the year 2020.(4)

In India, 0.11 million deaths occurred due to road traffic crashes in 2006, which is nearly 10% of the total road traffic deaths in the world.(5) Men aged less than 25 years are nearly three times as likely to be killed in road traffic crashes as compared to women(6) of that age. Pedestrians and bicyclists account for 70% of the road deaths, while 25% occur among motorized two wheeler riders. A large percentage of injuries go unreported due to lack of a systematic injury information system. Good quality information on mortality and morbidity, road design, and enforcement practices is essential for addressing the problem and for effective intervention strategies. Information on each of these is available in fragments from multiple agencies that deal with them. Realizing the need to improve the data on injuries, the Indian Council of Medical Research (ICMR) conducted this project to test the feasibility of a module to extract data on injuries from different sources in a prospective manner. This project came as a recommendation from the ICMR-WHO collaborative workshop on the ‘Development of a Feasibility Module on Road Traffic Injuries,’ in Delhi, in 2006.(7)

## Materials and Methods

### Hospital participation

The study was undertaken from April to September, 2007, by the Department of Epidemiology, NIMHANS, Bangalore, and the Department of PSM, BJMC, Pune. A letter of appeal to participate in the program was sent by ICMR to all hospitals in the two cities. About 23 hospitals in Bangalore and 12 hospitals in Pune responded to the appeal and agreed to participate in the study.

A series of stakeholder meetings were held with doctors, medical superintendents, police nodal persons, emergency care medical personnel, and nurses, along with the transport department and other non-governmental organizations. The purpose of these meetings was to apprise the participants of the importance of the study and share their data collection format. Various methods of linking the data collection formats and feedback mechanisms were discussed and a written consent was obtained for data sharing. Inventory details regarding the staffing pattern, number of injury victims, preparedness for a long-term program, and reasons for non-compliance, were gathered from these hospitals.

### Data collection

A questionnaire was developed and pilot tested on a few patients. The information collected consisted of personal identification data, date, and time of injury, nature, type, and place of injury, protective gear worn, mode of transportation, treatment management, and outcome. *No ICD-9 E-Codes or ICD-10 VWXY Codes were used*. The outcomes were classified into four categories, namely (i) Recovered and improved, (ii) Not recovered, (iii) Referred to another hospital, and (iv) Dead. The severities were broadly classified into:

Mild: Injuries that do not require hospital admission such as, abrasion, laceration, and so on.

Moderate: Injuries that require hospital admission and/or a stay in casualty for more than six hours like fractures, moderate external or suspected internal bleeding, large open wounds, where there is suspected injury to the internal organs and vitals are stable.

Severe: Injuries that require hospital admission and swift management like massive internal or external bleeding; cerebral hemorrhage, and vitals are not stable.

Training programs for the hospital staff (doctors, nurses, resident medical officers, and medical superintendents) and the representatives from traffic and transport were conducted using instruction manuals for completing the forms. The medico-legal records and case sheets were assessed for collecting additional information and for cross-checking. Injury data for six months (April 2007-September 2007) were collected on the prescribed form by the project staff for the first three months and by the regular hospital staff for the next three months. Data from the two centres were analyzed at the Division of Non-Communicable Diseases, ICMR using SPSS 15.0.

## Results

### Hospital characteristics

In Bangalore 21 urban hospitals and two rural hospitals, in Tumkur, participated in the study. In Pune 12 hospitals with more than a 100-bed strength participated in the study. Over 80% of these hospitals were private/private teaching hospitals. The inclusion of these hospitals was purely voluntary and together they catered to more than 70% of the injured patients from the city of Bangalore and Pune, respectively. The Hospital Information System in most of the hospitals was computerized. The largest hospital in Pune was the Sassoon Hospital that registered a total casualty load of 25,232 cases during the study period of six months (April-September, 2007). Of these 9122 (36.0%) were injuries of various types. In Bangalore, the Bhagwan Mahavir Jain Hospital registered the largest load of casualty cases (430-450 patients per week), but the load of injury cases was more in NIMHANS (180-200 per week) followed by Bowring and Lady Curzon Hospital (150-160 per week). In Pune 44 out of 56 doctors, 38 out of 85 nurses, and 25 residents were trained. In Bangalore 500 similar hospital staff were trained in 21 training programs. Two of the 12 hospitals in Pune showed high level of interest and were proactive, while seven were active initially, but gradually lost interest and three hospitals participated on demand. In Bangalore, the level of interest and cooperation from the hospitals was partial, as they had received no administrative orders.

The review meetings and training programs highlighted the need for coordination and integration of the Police, Transport, and Health Departments. Data were collected mainly for medico-legal purposes and was inadequate for policy development and program implementation. These departments lacked the capacity to systematically analyze and interpret the data. They did not have the time to collect the details and had the tendency to fall back on the existing system in the absence of a continuous monitoring system.

### Pattern of injuries

Data on 32,188 hospital registered injury cases could be extracted into the questionnaire prepared for this purpose. It took 8-10 minutes to complete the questionnaire. There were 24370 (76.9%) males and 7301 (23.1%) females. Children and young adults less than 29 years of age comprised 53% of these injuries, with a peak in 20-29 years. Road traffic crashes accounted for 44% of the injuries in both the cities. Injuries due to assaults were the second largest (25.3%) [Table 1] accounting for 19.4% of the injuries in Bangalore and 35% in Pune. RTI was most frequent in the age group of 15-44 years (71.4%).

**Table 1:** Cause of injury among different age groups

Age-group	<10	10-19	20-29	30-39	40-49	50-69	> = 70	Total
Road Traffic Injury	451 (3.6)	1267 (10.1)	4578 (36.6)	2700 (21.6)	1730 (13.8)	1568 (12.5)	226 (1.8)	12520 (42.8)
Fall	411 (13.6)	442 (14.6)	683 (22.6)	495 (16.4)	418 (13.8)	447 (14.8)	124 (4.1)	3020 (10.3)
Assault	30 (0.4)	689 (9.3)	2890 (39.2)	1997 (27.1)	1080 (14.6)	635 (8.6)	59 (0.8)	7380 (25.3)
Stab/cut	11 (2.0)	87 (15.8)	276 (50.3)	92 (16.8)	63 (11.5)	19 (3.5)	1 (0.2)	549 (1.9)
Burns	108 (10.2)	154 (14.5)	419 (39.5)	227 (21.4)	89 (8.4)	54 (5.1)	9 (0.8)	1060 (3.6)
Poisoning	89 (4.0)	424 (18.9)	1049 (46.8)	422 (18.8)	145 (6.5)	109 (4.9)	5 (0.2)	2243 (7.7)
Animal bite	159 (15.7)	205 (20.3)	205 (20.3)	184 (18.2)	120 (11.9)	119 (11.8)	18 (1.8)	1010 (3.5)
Fall of object	30 (5.7)	78 (14.9)	206 (39.5)	89 (17.0)	70 (13.4)	46 (8.8)	3 (0.6)	522 (1.8)
Others	59 (6.4)	127 (13.8)	317 (34.5)	186 (20.3)	125 (13.6)	92 (10.0)	12 (1.3)	918 (3.1)

Total	1348 (4.6)	3473 (11.9)	10623 (36.4)	6392 (21.9)	3840 (13.1)	3089 (10.6)	457 (1.6)	29222*

Figures in parentheses indicate percentages;

* Age groups/cause not recorded in 2966 cases

**Figure 1:**
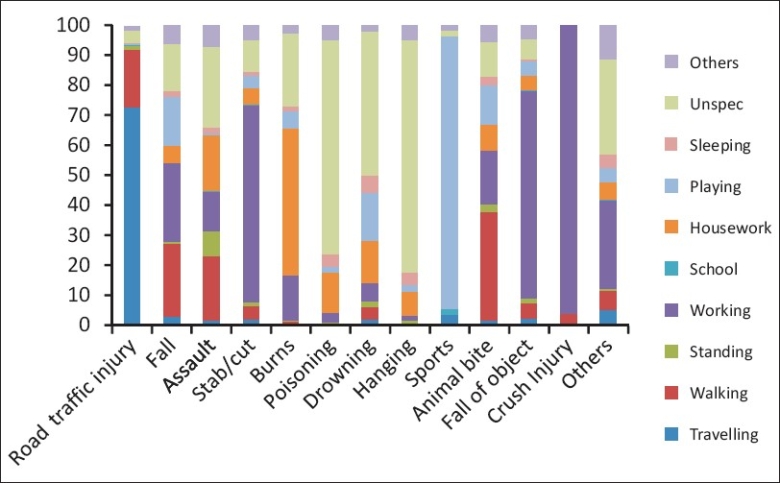
Activities at the time of injury

Most of the road traffic victims (73%) were injured when traveling in a vehicle, 19.0% got injured while walking on the road, and the rest (8%) while standing or working on the road. Among those who got injured while walking, 46.7% were victims of Road Traffic Injury and 30% were injured due to assaults. Assault was the main cause of injury for those who were standing (68.6%) or sleeping (30%). Majority (60%) of the injuries due to fall of object or stab/cuts occurred when the victim was working at home or at place of work. More than 70% of the cases injured due to poisoning and hanging did not mention the activity [Figure 1].

Among the road user categories, two-wheeler motorized riders were the most frequently injured, accounting for 28.6% of the Road Traffic Victims. This percentage was significantly more in Pune (33.3%) as compared to Bangalore (26.5%). A majority of them (66%) belonged to the age group of 15-34 years, while 17% were in the age group of 35-44 years. The other vulnerable road users were the pedestrians (23%). While pedestrians of all age groups were equally vulnerable on roads, a large proportion belonged to the age group of 25-34 years (18.3%). Pedal cyclists constituted 16.2% and two wheeler pillion riders about 10.0% of all the road traffic victims [Table 2].

**Table 2:** Distribution of road user types

Center Road User type	Bangalore		Pune		Total	
	n	%	*n*	%	*n*	%
Pedestrian	2438	(24.8)	789	(18.4)	3227	(22.8)
Pedal cyclist	2114	(21.5)	159	(3.7)	2273	(16.1)
Two-wheeler motorized rider	2606	(26.5)	1427	(33.3)	4033	(28.6)
Two-wheeler pillion	869	(8.8)	548	(12.8)	1417	(10.0)
Three-wheeler driver	121	(1.2)	98	(2.3)	219	(1.6)
Three-wheeler occupant	159	(1.6)	97	(2.3)	256	(1.8)
Car driver	110	(1.1)	75	(1.7)	185	(1.3)
Car occupant	281	(2.9)	125	(2.9)	406	(2.9)
Bus driver	115	(1.2)	42	(1.0)	157	(1.1)
Bus occupant	185	(1.9)	127	(3.0)	312	(2.2)
Truck driver	16	(.2)			16	(.1)
Truck occupant	54	(.5)			54	(.4)
Other four-wheeler driver	11	(.1)	50	(1.2)	61	(.4)
Other four-wheeler occupant	117	(1.2)	124	(2.9)	241	(1.7)
Unknown	523	(5.3)	574	(13.4)	1097	(7.8)
Others	119	(1.2)	52	(1.2)	171	(1.2)

Total	9838	(100.0)	4286	(100.0)	14125	(100.0)

**Figure 2:**
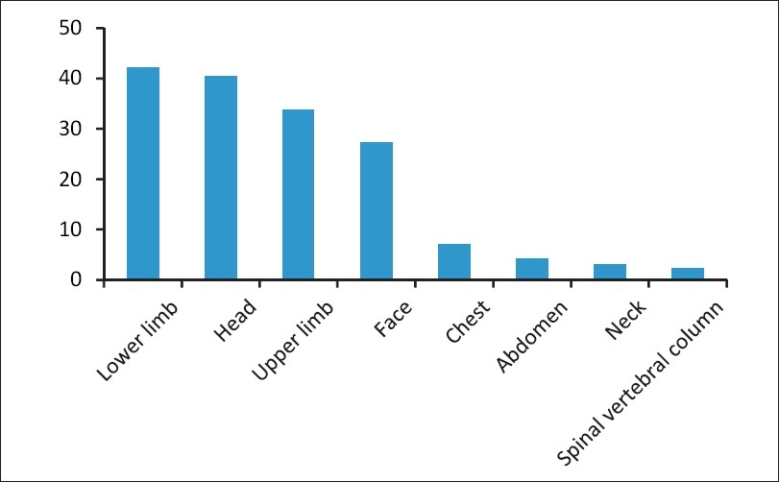
Major body parts injured among road traffic victims

Among the road traffic victims, 42.1% of the injuries affected the lower limbs. Upper limb injuries were reported by 33.7% victims, while head injuries occurred in 40.0% cases. Face injuries occurred among 27% cases. The upper and lower limbs and head injuries were the most frequent among the two wheeler riders. Among them head and face injuries occurred more among those who were not wearing helmets (61 and 36%) as compared to those who were wearing helmets (16.3 and 16%) [Figure 2].

Most of the victims (89%) of road traffic crashes were conscious at the time of admission to the hospital. Only 1% (137) were brought dead and 6.4% were unconscious. Among those who were injured on municipality roads 41% received first aid. This proportion was less compared to 64% victims on highways and 75% victims on rural roads who received first aid. Most of the injured (97%) were treated at the primary level by doctors.

About one-half of the injured (48%) reached the hospital on their own, while more than one-fifth were referred by government hospitals and 10.3% were referred by private hospitals. Only 19% of the victims were transported by ambulances. Forty-four per cent came on private vehicles or taxis and 28% on auto rickshaws. Police vans were very rarely used (2.3%). Majority (59%) of the victims were brought to the hospital by family members, while 25% were shifted to the hospital by acquaintances. Of the victims of road crashes who were transferred to the hospitals, 35% were treated and sent home, while 46% were admitted for medical and surgical care. One-fifth of the injured were treated in the Emergency Room and referred to another hospital.

The outcome data were available for only 6004 (42.5%) victims. Of these 4462 (74.5%) recovered and improved, in 19% the condition worsened, while 140 (2.3%) died. Severe injuries resulted in a worse outcome. Among mild injuries, more than 90% recovered, while among severe injuries only 51% recovered, in 31% the condition worsened, 8% died, and the rest were shifted to another hospital.

## Discussions

Enormous resources are spent on acute and long-term care of the injured. However, due to lack of good quality data on their geographical distribution and on the contributing factors, there is still a lot to be done in terms of their prevention and control. One impediment in this effort is the multi-sectoral nature of this problem. As road traffic injuries involves persons, vehicles, and roads, their prevention also involves cooperation from automobile manufacturers, road engineering, urban planning, and enforcement agencies, insurance and of course the health sector. In fact the health sector bears the brunt of all this, as the injured has to be treated and taken care of in the hospital. Hence, it is important for the health sector to take the lead, working in close cooperation with the other agencies. This study is the first step in this direction.

The study adopted a simple questionnaire that extracted only the core minimum data on the injuries, based on the WHO guidelines.(8) No efforts were made to exclude the non-serious cases. As most of the participating hospitals were not using ICD codes, it was thought that personal information of the victims, place, type of injury, and specific details of the road traffic injuries would be collected in the first instance. The injury severity was not assessed by using the Injury Severity Scoring. The two centers were able to encourage the hospital authorities for participation by training and orientation programs. Most of the emergency unit staff was trained for data retrieval from the emergency records in a prospective manner. When compared to the retrospective approach (largely dependent on the hospital information system) that was also being pilot-tested concurrently in five hospitals(9) in another study, the prospective approach yielded less missing data.

Both the studies have shown that while it is desirable to have a prospective approach that will provide a completely tailored data, with quality control and completeness in outcomes, it is going to involve higher costs, time, and resources. In contrast, the retrospective approach takes lesser time and is cost effective, but there is lack of quality control and it yields incomplete data. A model that appropriately knits both the approaches would be ideal.

The hospital-based data shows a pattern very similar to that reported by CDC in USA(10) with 64% unintentional injuries, 24% intentional, and 8% suicides. This study, like many other studies in India, has shown that more injuries occur in the 15-44 years age group, which are the most productive years of life. Similar observations have been made by two other studies(11,12) that have reported injuries in the 20-40 years age group.

Males are predominantly more (77%) in number than females as is also seen in a number of studies. Road traffic injuries constitute 44% of the total number of injuries. This is slightly more than the national figure of 34% in 2006. Assaults are only slightly less (39%). This indicates that injuries due to violence are no less than traffic injuries.

As observed in many other studies, the most vulnerable road users were pedestrians and two wheeler riders. Less than 25% of the victims reported wearing helmets or seat belts. Severity of injuries was more pronounced in non-users. Enforcement on these two aspects was therefore very essential. Injuries to the upper and lower limbs, head and face were most common as observed in many other studies.(11–13) Most of the injured were taken to the hospital in private vehicles and taxis. Ambulance was used only in 19% cases. This was slightly better than the observations in another study in Delhi in 2002, in which most of the accident victims were transported to the hospital in auto rickshaws and taxis (36%), two wheelers (2.1%), and ambulances (4.9%) [Table 3].(14)

Sustaining the system required additional incentives, infrastructure build-up, and a periodical orientation/training program. Both the inter- and intra-hospital data linking mechanisms had to be developed for such a system to be in place.

The study had its limitations, in that it was a hospital-based study that did not provide the burden in terms of prevalence of all injuries in an area. However, this method could be a beginning to the development of a database of serious injuries and deaths, in the absence of a surveillance system. Once this was in place, it would be possible to strengthen the database with parallel epidemiological studies that would provide disease-burden estimates. The findings from the study have given significant leads for initiating an injury surveillance program. The major areas that need to be addressed:

- Budgetary allocation for sustainability,
- Creating a central agency capable of guiding, coordinating, implementing, and monitoring the injury prevention program,
- Promoting training, capacity development, and feedback mechanism,
- Improving the hospital information system using state-of-the-art systems, and
- Dissemination and proper utilization of information for prevention programs.

**Table 3:** Mode of transportation of all the injured victims

Mode of transport	Ambulance		Government vehicle		Private vehicle or taxi		Auto rickshaw		Police van		Walking		Others		Total
Type of injury	N	%	N	%	N	%	N	%	N	%	N	%	N	%	N

Road traffic injury	1905	19.5	342	3.5	4247	43.6	2679	27.5	247	2.5	21	0.2	306	3.1	9747
Fall	348	15.0	114	4.9	642	27.6	993	42.8	62	2.7	12	0.5	151	6.5	2322
Assault	314	5.2	452	7.4	1826	30.0	2810	46.2	197	3.2	42	0.7	445	7.3	6086
Stab/cut	39	8.4	20	4.3	171	36.9	163	35.1	6	1.3	1	0.2	64	13.8	464
Burns	157	26.8	20	3.4	205	35.0	176	30.0	12	2.0	2	0.3	14	2.4	586
Poisoning	298	18.7	49	3.1	487	30.5	700	43.9	30	1.9	5	0.3	27	1.7	1596
Drowning	17	42.5	2	5.0	8	20.0	10	25.0					3	7.5	40
Hanging	28	32.6	5	5.8	20	23.3	26	30.2	5	5.8			2	2.3	86
Sports	9	17.6	5	9.8	9	17.6	23	45.1					5	9.8	51
Animal bite	86	9.1	162	17.2	424	44.9	221	23.4	8	.8	19	2.0	24	2.5	944
Fall of object	57	15.0	23	6.1	128	33.8	139	36.7	8	2.1	3	0.8	21	5.5	379
Crush injury	1	2.9	2	5.7	18	51.4	13	37.1			1	2.9			35
Others	87	28.8	15	5.0	72	23.8	93	30.8	16	5.3			19	6.3	302

Total	3346	14.8	1211	5.3	8257	36.5	8046	35.5	591	2.6	106	0.5	1081	4.8	22638
